# Liver perivascular epithelioid cell tumor in a patient with systemic lupus erythematosus

**DOI:** 10.1016/j.ijscr.2018.10.063

**Published:** 2018-11-01

**Authors:** Paraskevi V. Voulgari, Vissaria Tatsi, Haralampos J. Milionis, Anna Goussia, Vasileios Xydis, George K. Glantzounis

**Affiliations:** a Rheumatology Clinic, Department of Internal Medicine, Medical School, University of Ioannina, Ioannina, Greece; b Department of Surgery, Medical School, University of Ioannina, Ioannina, Greece; c Department of Internal Medicine, Medical School, University of Ioannina, Ioannina, Greece; d Department of Pathology, Medical School, University of Ioannina, Ioannina, Greece; e Department of Radiology, Medical School, University of Ioannina, Ioannina, Greece

**Keywords:** Neoplasm, Ischemia, Liver perivascular epithelioid cell tumor, Systemic lupus erythematosus, Thrombosis, Eosinophilia

## Abstract

**Introduction:**

Perivascular epithelioid cell tumor (PECOMA) is a rare mesenchymal neoplasm which expresses both myogenic and melanocytic markers showing a benign course,although malignant tumors have also been reported. To date there are approximately 33 cases of published hepatic pecomas.

**Presentation of case:**

We describe a 47-year-old man with a 27-year past medical history of systemic lupus erythematosus (SLE) who underwent left liver lobectomy due to a liver pecoma. His postoperative course complicated with infection, thrombosis of hepatic artery and liver ischemia as well as drug fever.

**Discussion:**

Treatment protocol especially for hepatic PECOMA has not reached a consensus although surgical resection is the preferred therapy.

**Conclusion:**

This is the first case of coexistence of liver pecoma and SLE.

## Highlights

• PECOMA is a rare mesenchymal neoplasm which expresses both myogenic and melanocytic markers showing a variable course.
• Association of SLE with hepatic PECOMA is unknown.
• Surgical resection is the preferred therapy.

## 1 Introduction

Perivascular epithelioid cell tumor (PECOMA) is a rare mesenchymal neoplasm which expresses both myogenic and melanocytic markers [[1], [2], [3]]. PECOMA can arrive from many locations of the body such as kidney, pancreas, urinary bladder, uterus and liver.

The clinical and histological characteristics of PECOMA have yet to be fully documented. Treatment protocol especially for hepatic PECOMA has not reached a consensus although surgical resection is the preferred therapy. We describe, for the first time, a case of liver pecoma in a patient with systemic lupus erythematosus (SLE).

## 2 Presentation of case

**Fig. 1:**
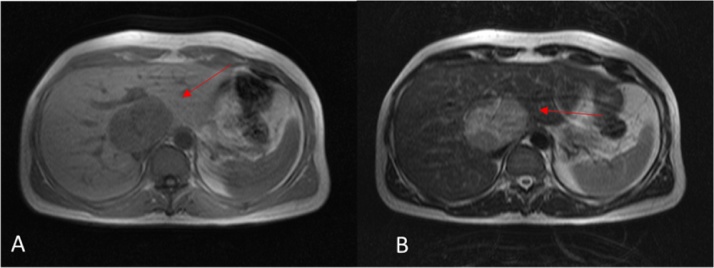
MRI of the abdomen. A well-marginated mass appears as a hypointense area on T1-weighted images (A), and hyperintense area on T2-weighted images (B).

A 47-year-old man with a 27-year past medical history of SLE was admitted to the Surgery clinic because of a liver mass 7 cm on computer tomography scan (CT) Fig. 1. SLE was diagnosed in 1990 based on, arthralgias, skin rash, lupus nephritis type IV (treated with cyclophosphamide) and positive antinuclear antibodies. SLE was complicated with deep vein thrombosis in his right leg in 1998 while antiphospholipid (APS) antibodies were reported negative at that time. SLE was inactive at admission and the patient was taking hydroxychloroquine. He underwent left hepatectomy with en block resection of segment I and cholocystectomy.

**Fig. 2:**
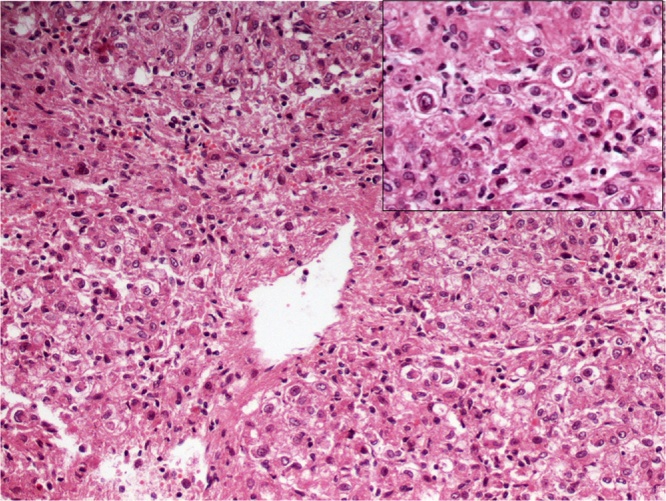
Nests and sheets of large cells with abundant eosinophilic to clear cytoplasm, round to oval nuclei and small nucleoli.

The histologic examination of the tumor revealed nests and sheets of large cells with abundant eosinophilic to clear cytoplasm, round to oval nuclei and small nucleoli with expression of HMB-45 and melan-A markers (Fig. 2) compatible with pecoma of uncertain malignant potential.

**Fig. 3:**
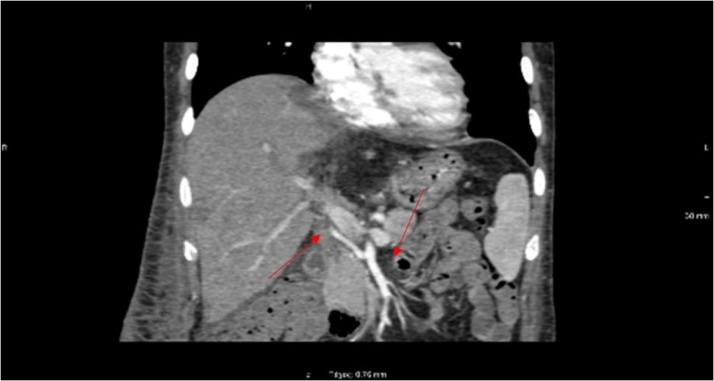
CTA of the abdominal aorta; Oblique maximum intensity projection image demonstrates the hepatic artery originating from the superior mesenteric artery. It also shows filling defect of the distal hepatic artery, followed by total occlusion.

Postoperative course (5th day) was complicated with fever up to 39o C with rigors, increased C-reactive protein (CRP)174 mg/L (normal<6) and white blood cells (WBC) 18,240 per cubic millimeter (reference range: 4000–11000). Blood and urine cultures were negative while infection with staphylococcus coagulase negative grew from drainage catherer. Antibiotics according to susceptibility testing were initiated. He remained afebrile until the 16th hospital day when fever without rigors presented. A moderate increase of CRP 58 mg/L and increase of aspartate aminotransferase (AST): 147 U/L(normal value <35) and alanine aminotransferase (ALT): 64 U/L (normal value <35) were found. A CT and magnetic resonance imaging (MRI) revealed an area with compromised blood supply compatible with tissue ischemia and/or abscess as well as thrombosis of hepatic artery(Fig. 3). The patient underwent resection of the ischemic-necrotic part of segment VIII. Full anticoagulation with low molecular weight heparin substituted for prophylactic dose and small doses of aspirin were introduced while APS antibodies remained negative.

At 27th hospital day while he was treated with vancomycin and imipenem the patient presented fever,normal WBC 5540 per cubic millimeter with eosinophilia(21% or 1263) and increased liver enzymes AST 83 U/L, ALT 59 U/L which were attributed to drug fever. Antibiotics were stopped while prezolon 0.5 mg/kg/day was initiated with symptom resolution. The patient left hospital treated with prezolon, anticoagulation and hydroxychloroquine. Eighteen months later his laboratory examination as well as abdomen MRI findings were unremarkable.

## 3 Discussion

Coexistence of malignancy and autoimmune rheumatic disease such as SLE may be linked with underlying pathophysiologic mechanisms which are not fully understood. Long-term and often severe immune stimulus of autoimmune disease has been associated to malignancy and on the other hand long-term suppression of the immune response with drugs is also connected to risk of subsequent neoplasm. SLE is associated with an overall increased risk of malignancy, particularly non-Hodgkin’s lymphoma, lung, liver, vulvar/vaginal and thyroid and a decreased risk of breast and prostate cancer. Viral reactivation and upregulation of cytokines such as B cell activating factor and interleukin 6 have been implicated in pathogenesis of both lupus and lymphomas [4,5]. Increased risk of hepatobiliary malignancies has been reported [4,6]. Viral hepatitis may account for the increased rate of primary liver cancer [7,8]. Drugs like cyclophosphamide used in SLE treatment may be a risk factor for later cancers [9]. Association of SLE with pecomas is unknown.

On the other hand, chronic inflammatory status, disease activity and accelerated atherosclerosis as well as antiphospholipid antibodies contribute to the thrombosis phenotype of SLE [10].

Hepatic pecomas are rare but increasingly recognized tumors. To date approximately 33 cases of hepatic pecomas have been reported [[1], [2], [3]].They can occur at any age, although it has been reported that they predominantly affect women aged 30–50 years old [11]. Hepatic PECOMA may occur as a solitary mass or as multiple lesions, which have been suggested to be associated with tuberous sclerosis. There are several hypotheses concerning the origin of hepatic PECOMAS. They may develop from undifferentiated neural crest cells which coexpress the phenotype of smooth muscles and melanocytes or they derive from local pericytes or they have smooth muscle origin [12,13]. Microscopically epithelioid cells, spindled cells and adipocytes may be present. They radially arrange around the vascular lumen, exhibiting small centrally placed, normochromatic, round-to-oval nuclei with small nucleoli, although hyperchromasia and nuclear irregularity may be found. PECOMAS are positive for both melanocytic and muscle markers [14]. These immunological markers include HMB-45, Melan-A and smooth muscle a-actin. A recent review identified 33 cases of primary hepatic PECOMAS from 25 articles [15].

Patients are often asymptomatic. Gastrointestinal symptoms exist when the tumor size cause localized pressure effect [1]. Imaging diagnosis can be challenging because the quantity of adipose tissue, irregular vessels and smooth muscle cells is various. Hepatic PECOMA is often misdiagnosed as carcinoma, hemangioma or other liver malignancies. Nevertheless, correct diagnosis is possible in patients with an asymptomatic liver tumor and normal serological test results. Surgery is indicated in patients presenting with large tumors (>5 cm), progressive enlargement or malignant tendency [1].

## 4 Conclusion

We described for the first time a patient with hepatic pecoma, a rare mesenchymal tumor and SLE who was successfully treated with tumor surgical resection. Long term clinical follow-up is necessary in patients with hepatic pecomas because the nature of the disease is not entirely known at present.

## Conflicts of interest

The authors have nothing to declare.

## Funding

The authors have nothing to declare.

## Ethical approval

The study is exempt from ethnical approval in our institution.

## Consent

Written informed consent was obtained from the patient for publication of this case report and accompanying images. A copy of the written consent is available for review by the Editor-in-Chief of this journal on request.

## Author contribution

Paraskevi V. Voulgari: **Writing - original draft**

Vissaria Tatsi: **Resources**

Haralampos J. Milionis: **Resources**

Anna Goussia: **Resources**

Vasileios Xydis: **Resources**

George K. Glantzounis: **Writing - review & editing**

## Registration of research studies

The authors have nothing to declare.

## Guarantor

***Paraskevi V. Voulgari***, Professor of Rheumatology, Rheumatology Clinic, Department of Internal Medicine, Medical School, University of Ioannina, Ioannina, Greece, pvoulgar@cc.uoi.gr, www.rheumatology.gr, tel.: +302651099832, fax.: +302651046617.

## Provenance and peer review

Not commissioned, externally peer reviewed.
